# Hypomethylating agents are the current therapy backbone for MDS and AML relapsing after allogeneic hematopoietic stem cell transplantation

**DOI:** 10.3389/fimmu.2026.1895791

**Published:** 2026-07-22

**Authors:** Guido Kobbe, Titus Watrin, Felicitas Schulz, Annika Kaprzak, Kathrin Nachtkamp, Ulrich Germing, Sascha Dietrich, Paul Jäger

**Affiliations:** Department of Hematology, Oncology and Clinical Immunology, University Hospital Düsseldorf, Heinrich Heine University, Medical Faculty, Düsseldorf, Germany

**Keywords:** allogeneic stem cell transplantation, azacytidine, decitabine, donor lymphocyte infusions, hypomethylating agents, relapse, second transplant

## Abstract

Relapse of myelodysplastic syndrome (MDS) and acute myeloid leukemia (AML) is associated with poor prognosis and remains the most common cause of treatment failure following allogeneic hematopoietic stem cell transplantation (alloHSCT). Over the past two decades, hypomethylating agents (HMAs), particularly azacytidine, have evolved from an experimental approach to the current therapeutic backbone of relapse therapy following alloHSCT, although future treatment paradigms may further evolve with emerging targeted and immunotherapeutic approaches. The RELAZA studies demonstrated that preemptive therapy with azacytidine in molecular relapse can significantly delay the onset of hematologic relapse. However, sustained disease control generally requires additional immunological mechanisms. Against this backdrop, the combination of azacytidine—either alone or in combination regimens—with donor lymphocyte infusions (DLI) has been established as a successful salvage strategy. Numerous studies have consistently shown over the years that a low disease burden, molecular rather than hematological relapse, and a longer interval between transplant and relapse are associated with better response rates and prolonged survival. This underscores the great importance of MRD monitoring for enabling early intervention. Prognostic models such as the APSS-R score incorporate these factors. Additionally, the score takes into account pre-transplant therapy, thereby underscoring the importance of a biologically tailored treatment strategy to prevent the emergence of treatment-resistant clones. Recent combination therapies with targeted agents, such as FLT3 and IDH inhibitors, as well as with venetoclax or lenalidomide, are showing promising results. Future strategies will likely focus on individualized, biological1ly guided treatment approaches and a selective indication for second alloHSCT.

## 1 Introduction

Despite significant advances in transplant medicine, relapses of MDS and AML remain the most common cause of treatment failure following alloHSCT. Depending on the biology of the disease, most relapses occur either early, typically within the first year after transplantation (predominantly AML and CMML), but also in about 30% of patients later during follow-up. The latter is typical for less proliferative AML and MDS (1, 2). Traditionally, the treatment of relapse following alloHSCT depended on the patient’s clinical condition and involved intensive salvage chemotherapy or low-dose Ara-C —often combined with the administration of donor lymphocyte infusions (DLI), DLI alone or a second alloHSCT. Particularly in cases of late-onset relapse—defined as recurrence occurring more than twelve months after the initial transplantation—a second alloHSCT was historically considered the preferred treatment strategy, provided that disease control could be re-established with conventional chemotherapy. These therapeutic approaches were characterized by modest activity but high toxicity. Sustained remissions or even cures were generally rare with these strategies (3, 4).

A major impetus for the development of less toxic immunomodulatory therapeutic approaches came from the initial report by Graef et al. in 2007, which described for the first time the successful treatment of an AML relapse following alloHSCT using azacytidine in combination with DLI (5). In the years that followed, this approach increasingly established itself as the preferred treatment option for relapse following alloHSCT. Several phase II studies—most of which were single-arm or retrospective trials—studied the use of hypomethylating agents (HMA), particularly azacytidine, as monotherapy or in combination with DLI. In addition, various combination regimens were evaluated, including targeted agents such as sorafenib, enasidenib or ivosidenib, immunomodulatory agents such as lenalidomide, or the BCL2 inhibitor venetoclax as a partner to azacytidine.

In this review, we summarize the essence of the clinical studies published to date on HMA and combined immunomodulatory therapeutic approaches for relapsed MDS and AML following alloHSCT. Furthermore, we analyze clinical and biological prognostic factors associated with improved response and long-term therapeutic success in this high-risk setting.

## 2 Main Text

### 2.1 Causes of relapse following allogeneic hematopoietic stem cell transplantation

From a pathophysiological perspective, relapse following alloHSCT is primarily driven by clonal selection resulting from therapeutic and immune pressure. Molecular analyses have shown that relapses following alloHSCT are frequently associated with dysregulation of immune-relevant signaling pathways, particularly with a marked reduction in MHC-II expression or, for example, with complete HLA loss following haploidentical hematopoietic stem cell transplantation (6, 7). As a result, leukemic cells evade immune recognition by the donor’s T cells. These changes are partially reversible and suggest an adaptive mechanism of immune evasion. In addition, there are signs of functional T-cell exhaustion with activation of inhibitory signaling pathways such as PD-1/PD-L1, TIM-3, or TIGIT, accompanied by impaired cytokine production and reduced T-cell receptor diversity (8). NK-cell dysfunction is also thought to contribute to immune evasion. The occurrence of graft-versus-host disease (GvHD) is often a clinical sign of a graft-versus-leukemia (GvL) effect. However, GvHD is not a universally reliable surrogate marker for effective immunological control of the underlying disease in the sense of a sustained GvL effect. In particular, when using modern GVHD prophylaxis regimens with post-transplant cyclophosphamide or following initially successful relapse therapy, further, often extramedullary relapses may occur despite the presence of GvHD (9, 10).

Persistence is frequently observed, particularly of mutations in epigenetic regulators and CHIP-associated clones, while mutations in proliferation-promoting signaling pathways evolve more dynamically. The leukemic stem cell and early progenitor compartments appear to play a central role. Leukemic stem cells express various immune-escape molecules and benefit from protective interactions with the bone marrow niche (11). Studies in MDS have shown that residual stem cell populations are detectable long before morphological relapse and, in some cases, have already undergone clonal selection during prior therapy with hypomethylating agents (12). This supports the concept of quiescent, niche-protected residual clones as the source of later relapses. Additionally, immunosuppressive factors in the bone marrow microenvironment, including TGF-β1, IDO1, or regulatory macrophage populations, promote the survival of residual leukemic cells (13).

### 2.2 Hypomethylating agents

Hypomethylating agents (HMAs) are azanucleosides whose mechanism of action cannot be reduced to a single mechanism. Azacytidine and decitabine are the most clinically relevant agents for the treatment of MDS and AML. Guadecitabine, on the other hand, remains an investigational agent without a proven Phase III survival benefit. In Europe, azacytidine is the most established treatment for MDS, while decitabine and decitabine/cedazuridine are more widely used for MDS/CMML in North America. For AML, decitabine and decitabine/cedazuridine are approved in Europe for patients who are unsuitable for standard intensive induction therapy.

HMAs are prodrugs that have very different biological effects depending on dose, duration of exposure, and cell cycle. They must first be phosphorylated and incorporated during the S phase to become effective. After DNA incorporation, they form covalent adducts with DNA methyltransferases (DNMTs), leading to DNMT depletion, replication stress, and, over time, passive demethylation. At low doses, demethylating or modulating effects are primarily active, whereas at high doses, direct cytotoxic effects dominate (14).

Azacytidine and decitabine share this DNA-related core mechanism but are not biologically identical. A significant portion of azacytidine is incorporated into RNA, where it influences rRNA/tRNA processing, translation, and ribonucleotide reductase-dependent processes. Only a fraction enters the DNA via conversion. Decitabine, by contrast, is a more DNA-centric HMA. This explains why both substances can generate similar DNA markers such as DNMT1 depletion and hypomethylation, but trigger divergent transcription and cell cycle programs (15, 16).

HMAs also have immunological effects in clinical use that are quite ambivalent. Well-documented is the re-expression of tumor antigens and of programs relevant to antigen presentation. Azacytidine can increase the expression of MAGE antigens and induce a CD8 T-cell response against them. Similar data exist for other CTAs. Multi-omics analyses in AML cell lines also showed that azacytidine can alter not only the methylome but also the expression of surface antigens, resulting in the expression of immunologically relevant molecules on the cell surface (17, 18).

A second mechanism is “viral mimicry.” Demethylation and transcription of endogenous retroviral elements generate dsRNA-like signals that can activate type I interferon pathways and antiviral programs. This mechanism was initially described in epithelial tumor models but has since also been demonstrated in AML/MDS in translational studies (19–21).

T-cell effects of HMAs are context-dependent and sometimes contradictory. On the one hand, treatment with azacytidine is associated with a reconstitution of the bone marrow TCR repertoire, which was linked to improved survival, as well as an expansion of Treg cells alongside tumor-antigen-specific CD8 activation after alloHSCT. Following alloHSCT, Treg expansion during azacytidine therapy is well documented; in other therapeutic settings, however, a reduction in Treg proliferation capacity and a decrease in Th1/Th2 cells were observed during prolonged azacitidine treatment. On the other hand, azacytidine can also upregulate PD-1 in T cells and, in certain settings, promote a T-cell phenotype with a stronger inhibitory effect (22–27).

The effect of HMA on NK cells is described in similarly multidimensional terms. On the one hand, an “imprint” of azacytidine on the NK cell repertoire has been described, while other studies in AML and MDS patients showed impaired NK cell activity during therapy with azacytidine (28, 29). The evidence is also heterogeneous for myeloid-derived suppressor cells (MDSCs) and cytokines. In model systems, decitabine can deplete MDSCs and thereby break immune tolerance. However, this observation has not yet been established in clinical AML/MDS cohorts. The same applies to the cytokine environment in the bone marrow of patients with MDS and AML during therapy with HMAs (30–32).

In a clinical context, this means that HMA-induced immunomodulation is not automatically synonymous with greater anti-leukemic efficacy. Depending on the time window, compartment, combination therapy, and clinical situation, it can be beneficial, neutral, or even inhibitory to clinical success. In the specific context of treating relapse after alloHSCT, particularly in combination with DLI, the positive effects appear to outweigh the negative ones overall. Perhaps the most compelling argument for HMA therapy is the potential to attenuate GvHD without losing the graft-versus-leukemia effect. Experimental studies in mice have shown that azacytidine can attenuate experimental GvHD while simultaneously preserving GvL, due to differential effects on effector T cells and natural Tregs. In the clinical setting as well, following alloHSCT during relapse therapy with azacytidine, Treg expansion was observed within the first three months, accompanied by a cytotoxic CD8 response against tumor antigens such as MAGE, BAGE, and WT1 (23, 33, 34).

### 3.3 Azacytidine monotherapy

There are only a few studies in the literature in which azacytidine alone, without DLI, was used as relapse therapy following alloHSCT. In this regard, the RELAZA studies from Dresden therefore hold a special place. In these studies, DLI was not a primary component of the study design. The first RELAZA study (35) was a prospective, open-label, single-center Phase II study in adult patients with CD34+ AML/MDS following alloHSCT.

MRD was monitored in peripheral blood via chimerism analyses on selected CD34+ cells. A decline in donor chimerism below 80% in the CD34+ cell fraction in patients in hematologic remission was defined as MRD relapse. This triggered intervention with conventionally dosed azacytidine therapy (azacytidine 75 mg/m² s.c. on days 1–7 every 28 days). The primary endpoint was the proportion of patients who showed a significant response after completion of four azacytidine cycles (>80% CD34+ donor chimerism after 4 cycles). Secondary endpoints were: minor response, 6-month follow-up, and toxicity. Of 59 patients in the screening phase, 20 met the inclusion criteria. Overall, 10 of 20 patients achieved a major response (50%, 95% CI 27–73), 6 of 20 achieved a minor response (30%, 95% CI 12–54), and 4 of 20 relapsed during or shortly after the first 4 cycles. Overall, 13 of 20 patients (65%) experienced a hematologic relapse after a median of 231 days from the first MRD detection. After a median follow-up of 487 days, 8 of 20 patients (40%) were alive. Major toxicities were Grade 3/4 neutropenia and thrombocytopenia, which occurred in 80% and 65% of patients, respectively. *De novo* GHD was not observed. This study demonstrated that hematologic relapses can be substantially delayed, but usually not definitively prevented, by MRD-triggered therapy with azacytidine alone.

In the follow-up study RELAZA 2, the concept was expanded to include other MRD triggers and applied both to the scenario following intensive chemotherapy without alloHSCT and to a multicenter setting (36, 37). In the current publication from 2026, the researchers report on the long-term efficacy of MRD-guided preventive treatment with azacytidine for molecular relapse. For MRD detection, both RT-qPCR—mostly targeting the NPM1 mutation—and, following alloHSCT, chimerism analysis on selected CD34+ cells were employed. MRD relapse was defined as an NPM1 or RUNX1-RUNX1T1 level >1% or, after alloHSCT, donor chimerism of less than 80% in selected peripheral CD34+ cells. Patients who became MRD-positive were treated with azacytidine for up to 2 years. The long-term results of this study show that patients who achieve sustained MRD-negative remission have an excellent prognosis. At the same time, MRD relapse is an early sign of a much poorer clinical course. However, patients who achieved a second molecular response after treatment with azacytidine had significantly improved relapse-free survival and prolonged overall survival. Whether, in the setting following alloHSCT, cures are routinely achieved with azacytidine therapy alone in cases of MRD recurrence or whether this remains limited to individual cases remains an open question (36). **Table 1**.

**Table 1:** Key studies on Azacytidine +/- DLI for the treatment of relapse after alloHSCT.

Author	Year	Disease	Therapy **	Number	Response*	Main findings/conclusions
Platzbecker (35)	2012	AML/MDS	Azacytidine mono	20	Major 50% S**Table 3**0%	Pre-emptive Aza in MRD+ patients can delay hematologic relapse.
Platzbecker (36)	2026	AML/MDS	Azacytidine mono	95	Major 67%	Prolonged response >2years in 50% of responders, MRD negativity prolongs survival.
Czibere (39)	2010	AML/MDS	Azacytidine + DLI (82%)	22	ORR 72% CR 23%	Early response identifies long-term survivors, *de-novo* onset of aGvHD in 33%
Lübbert (40)	2010	AML/CMML	LD-Azacytidine + DLI (73%)	26	ORR 66% CR 16%	Low rate of *de-novo* aGvHD, objective responses also in poor risk cytogenetics.
Steinman (41)	2015	AML/MDS	LD-Azacytidine + DLI (90%)	72	ORR 44% CR 10%	Long-term remissions rare, feasible as an outpatient bridge to 2^nd^ transplant.
Schroeder (42)	2013	AML/MDS	Azacytidine + DLI (73%)	30	ORR 30% CR 23%	Better response with MDS and low disease burden AML, long-term remissions in 17%.
Schroeder (43)	2015	AML/MDS/MPN	Azacytidine + DLI (68%)	154	ORR 33% CR27%	Better prognosis in molecular relapse, MDS and low blast count AML. MDS 2-y OS 66%,
Craddock (44)	2016	AML/MDS	Azacytidine + DLI (38%)	181	ORR 25% CR 15%	Better prognosis with low disease burden and longer time to relapse. ARP-Score
Claiborne (45)	2019	AML/MDS	Azacytidine + DLI (100%)	28	ORR NA CR 36%	Low rate of GvHD, better response with molecular relapse and cGvHD.
Woo (46)	2017	AML/MDS	Azacytidine + DLI (3%) with relapse < day +100 after Tx	39	ORR 30% CR 8%	Response in early molecular relapse possible, pre-transplant therapy influences response.
Rautenberg (47)	2020	AML/MDS/MPN	Azacytidine + DLI (70%)	151	ORR 46% CR 41%	Better prognosis with low disease burden and longer time to relapse. APSS-R Score
Liberatore (48)	2022	AML/MDS	Azacytidine + DLI (46%)	71	ORR 49% CR 38%	Active in HLA-mismatch and haploidentical transplant, benefit of DLI, APSS-R score valid.
Karakulska (49)	2018	AML/MDS	Azacytidine + DLI (39%)	24	ORR 42% CR 30%	Better response with pre-emptive treatment than in open relapse, responses in complex karyotype.
Ozawa (50)	2019	AML/MDS	Azacytidine + DLI (23%)	22	ORR NA CR 32%	Better response and long-term survival in late and molecular relaps, benefit of DLI.

**Table 1** shows details of the most important studies in Azacytidine +/DLI for the treatment of MDS and AML relapse after alloHSCT. * Major, major response; Stable, stable disease; ORR, overall response (CR/CRi + PR); CR, complete response (CR, CRi). ** (…%) % of patients who received DLI.

### 3.4 Azacytidine and DLI

The combination of azacytidine and donor lymphocytes is currently believed to be the most commonly used salvage strategy in clinical practice for the treatment of relapsed MDS and AML following alloHSCT. In a recent survey among 14 German transplant centers, all reported that this combination is their preferred treatment option for relapses of myeloid neoplasms following alloHSCT (38).

Following an initial case report by Graef et al. in 2007, in which an early AML relapse was converted to long-term remission using the combination of azacytidine and donor lymphocytes, we first conducted a multicenter retrospective analysis at German transplant centers and identified 22 patients who had been treated with this therapeutic approach (5, 39). All patients had experienced hematologic relapse of AML (13) or MDS (8) a median of 103 days (57–708) after alloHSCT and received azacytidine at a dose of 100 mg/m² over five days. In addition, 82% of the patients received DLI. Overall, 72% of the patients showed a hematologic response to therapy, with five patients (23%) achieving complete remission. Acute GvHD occurred in 33% of patients following DLI. Early response after the first cycle was associated with improved long-term survival.

At the same time, Lübbert et al. published a cohort of 26 patients with AML (24) or CMML (2) who had received a reduced dose of azacytidine (100 mg absolute over 3 days) for the treatment of relapse following alloHSCT. In 73% of patients, azacytidine was combined with DLI. This analysis also demonstrated significant clinical activity, with a response in 66% of patients; 16% achieved complete remission. Overall, the toxicity of this therapeutic approach was low. These results of a low-dose azacytidine approach were later confirmed in a subsequent analysis by the same group. Approximately one-third of the patients subsequently received a second alloHSCT as consolidation therapy (40, 41).

**Figure 1:**
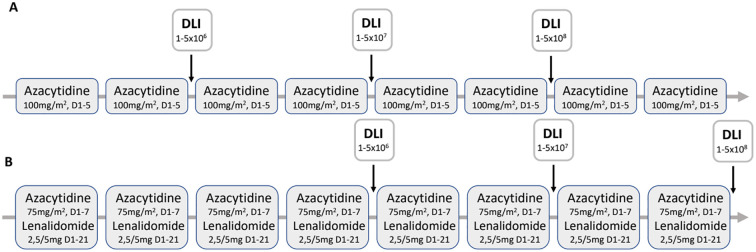
Study protocols for the AZARELA study **(A)** and the AZALENA study **(B)**. In combination therapy with early DLI, the cytotoxic and immunological effects of azacytidine and DLI cannot be separated. In the AZALENA study, however, 50% of remissions occurred before the first DLI administration. In clinical practice, however, the design of the AZARELA study **(A)** is preferred to achieve a faster response through early synergism (42, 69).

Building on these initial results, we conducted the first controlled, prospective, single-arm Phase II study for the treatment of AML and MDS relapse following alloHSCT with azacytidine and donor lymphocytes [**Figure 1**, (42)]. Thirty patients received up to eight cycles of azacytidine at a dose of 100 mg/m²/day on days 1–5 at 28-day intervals. After every two cycles, donor lymphocytes were administered in escalating doses ranging from 1–5×10^6^ to 1–5×10^8^ CD3-positive cells/kg. All patients had experienced a hematologic relapse at a median of 175 days (19–1,688) after alloHSCT. A median of 3 cycles of azacytidine (1–8) were administered, and 73% of patients received at least one DLI. The overall response rate was 30% and included seven complete remissions (23%), which persisted in the majority of patients until the end of the follow-up period of up to 3 years. The incidence of acute and chronic GvHD was 37% and 17%, respectively. Marked hematological toxicity was observed in 65% of patients, while infections represented the most common non-hematological toxicity.

As this therapeutic approach became increasingly established due to its good outpatient feasibility, comparatively low toxicity, and initially unexpectedly high efficacy, several retrospective multicenter analyses were published in the following years. In 2015, we published a retrospective analysis from 12 German transplant centers. A total of 154 patients with AML (124), MDS (28), or myeloproliferative neoplasms (MPN) (2) were treated with a median of four cycles of azacytidine. Additionally, 68% of patients received a median of two (1–7) donor lymphocyte infusions. The overall response rate was 33%, comprising 27% complete remissions and 6% partial remissions. In multivariate analysis, molecular relapse and a diagnosis of MDS emerged as the strongest predictors of achieving complete remission. A correlation between treatment response and disease burden was also observed in patients with AML. Accordingly, the 2-year survival rate was significantly higher in patients with MDS than in patients with AML and hematologic relapse. This study demonstrated for the first time a direct correlation between disease burden and the effectiveness of this therapeutic approach (43).

**Figure 2:**
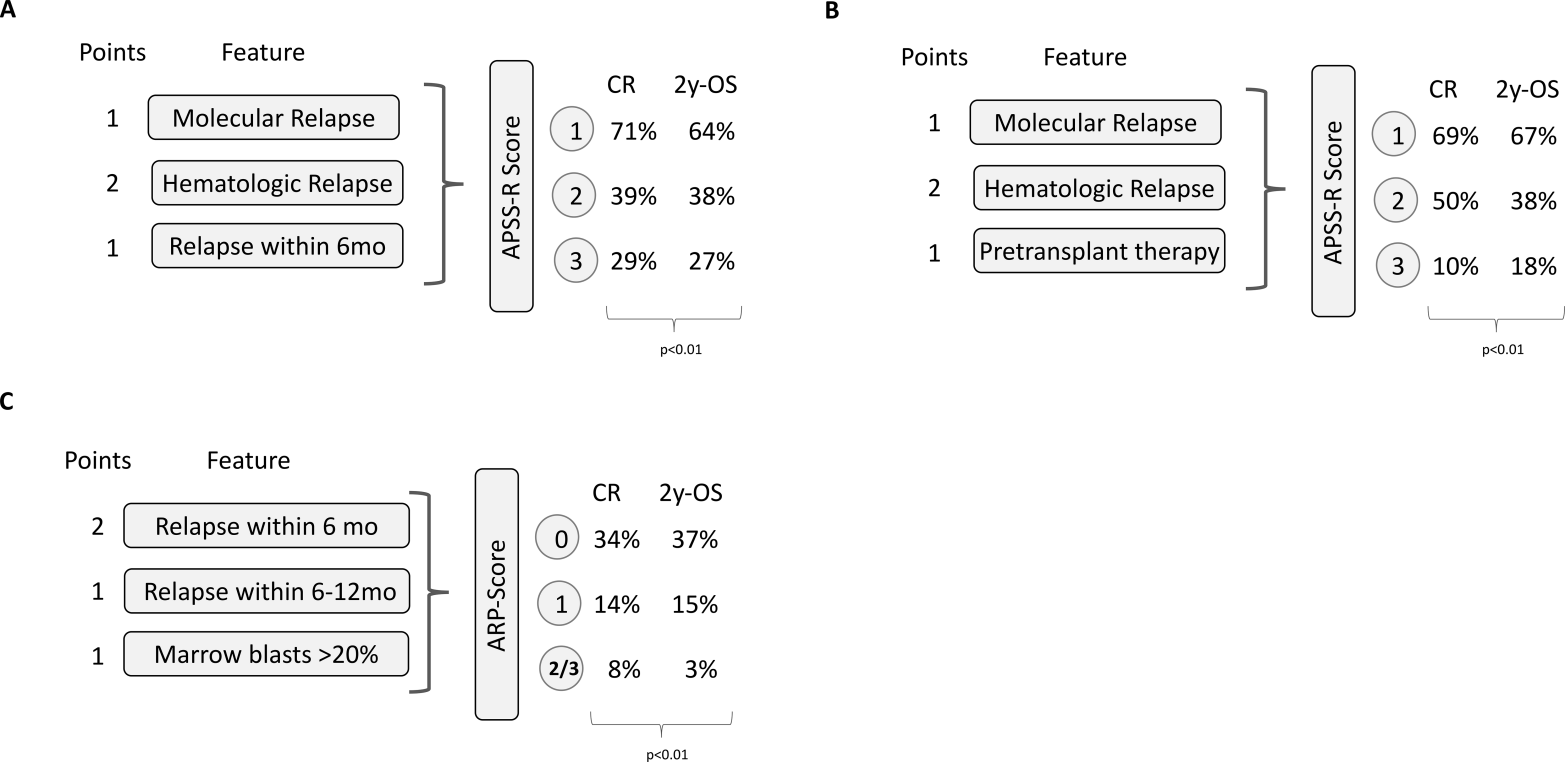
**(A)** The APSS-R Score according to Rautenberg et al. (47) was developed with data from 151 patients with MDS, sAML and *de-novo* AML with relapse after alloHSCT, who were treated with Azacytidine. Patients with molecular relapse were included. Seventy percent of patients DLI. **(B)** The APSS-R Score for MDS and sAML according to Rautenberg et al. (47) was developed with data from a subgroup of 66 patients with MDS and sAML excluding *de-novo* AML with relapse after alloHSCT, who were treated with Azacytidine and DLI. Here pre-transplant therapy is an adverse prognostic factor for response to salvage therapy and survival. **(C)** The ARP Score according to Craddock et al. (44) was developed with data from 181 patients with MDS and AML and hematologic relapse after alloHSCT, who were treated with Azacytidine. Patients with molecular relapse were not included. Only 38% of patients received DLI.

Shortly thereafter, Craddock et al. published an EBMT analysis of 181 patients with AML (116) or MDS (65) who had been treated with azacytidine within one month of the diagnosis of hematologic relapse following alloHSCT. In addition, 38% of patients had received at least one DLI. Overall, 25% of evaluable patients responded to therapy, and 24 patients (15%) achieved complete remission. Higher response rates were observed in patients who had been transplanted while in complete remission, as well as in patients with MDS. Patients who achieved complete remission had a 2-year overall survival rate of 48%, compared with 12% in the overall population. In the multivariate analysis, time to relapse and disease burden, as measured by blasts in the bone marrow, were identified as key prognostic factors for overall survival. Based on these parameters, the authors developed a prognostic (APR) score to predict survival (**Figure 2**). However, this analysis could not demonstrate a significant survival benefit from the additional administration of donor lymphocytes, and it also remained unclear how many patients additionally received a second allogeneic hematopoietic stem cell transplant (44).

A retrospective American analysis of 28 patients also showed that a low disease burden in the sense of molecular or cytogenetic relapse, as well as the occurrence of GvHD during the course of therapy, were associated with improved survival. In this study, overall survival was 44% one year after the start of therapy and 35% after two years (45). A prospective American study included 39 patients with AML or MDS who had relapsed within 100 days of alloHSCT. In the majority of cases, the relapse was diagnosed at a low disease burden using molecular genetic, flow cytometric, or cytogenetic methods. Patients received standard therapy with azacytidine and, following tapering of immunosuppression, optional DLI. The overall response rate was 30% and included three complete and nine partial remissions; an additional three patients achieved stable disease. Furthermore, there were indications that the type of pre-transplant therapy might influence response and overall survival. Patients who had received intensive prior therapy had a more favorable prognosis than patients who had undergone standard induction or therapy with hypomethylating agents or lenalidomide (46).

In a retrospective analysis of 151 patients from our center with relapsed myeloid neoplasias following alloHSCT, we systematically reviewed the prognostic factors identified to date. Of these, 61% of patients had a hematologic relapse and 39% had a molecular or cytogenetic relapse. Patients received a median of five cycles of azacitidine, and 70% received at least one donor lymphocyte infusion, which was administered according to the clinical course, including successful tapering of immunosuppression and the absence of clinically relevant GvHD. The overall response rate was 46%, with 41% of patients achieving complete remission. This was achieved after a median of four treatment cycles and lasted a median of 11 months. Consistent with the concept of disease burden as a major determinant of treatment success, patients treated for molecular or cytogenetic relapse achieved significantly higher response rates and superior survival compared with patients with hematologic relapse (CR rate 61% vs. 28%, respectively; 2-year OS 54.6% vs. 29.0%, respectively). The 2-year survival rate was 38%, and 17 patients showed sustained remissions of more than five years. At the time of analysis, some patients had already been in sustained remission for more than ten years. No patient had received a consolidating second alloHSCT while in remission.

In the multivariate analysis, disease burden at the time of relapse and the time from transplantation to relapse proved to be significant prognostic factors. Based on these variables, we developed a prognostic score (APSS-R) that could accurately predict patient survival. For the subgroup of MDS patients, it was also found that patients who had undergone transplantation upfront—that is, without prior therapy—responded significantly more frequently to relapse therapy than patients who had received prior treatment, such as with hypomethylating agents (47).

A German-Italian consortium subsequently conducted a retrospective analysis of 71 patients with AML or MDS who had experienced an MDS or AML relapse a median of nine months after alloHSCT from alternative donors, including mismatched unrelated donors (55%), haploidentical donors (41%), and mismatched cord blood (4%). All patients received azacytidine, and 46% were additionally treated with DLI. After a median of four treatment cycles, the overall response rate was 49%, and 38% achieved complete remission, which lasted a median of 17 months (8–89). The incidence of acute and chronic GvHD was 27% and 18%, respectively. The 1-year overall survival rate was 53%. Treatment of molecular relapse was also significantly more successful in this study than treatment of hematologic relapse (65% versus 15%), which was also reflected in a significantly higher 1-year survival rate (79% versus 34%). Furthermore, it was demonstrated that the additional administration of donor lymphocytes was associated with higher response rates as well as prolonged event-free and overall survival. The APSS-R score also allowed for reliable prognostic stratification in this cohort (48).

Parallel to and following the studies already mentioned, numerous, predominantly smaller retrospective analyses from individual centers or smaller consortia were published, without yielding any significant new insights regarding the prognostic and therapeutic factors already described (49, 50).

Finally, in a meta-analysis of 13 studies involving 811 patients, Li et al. summarized the pooled response rates and survival data and conducted subgroup analyses to identify prognostic factors. They found that the combination of azacytidine and DLI led to remissions in a significant proportion of about one-third of patients, and long-term survival was also achieved in about one-third of patients. The rates of acute and chronic GvHD were moderate overall. Adverse cytogenetics and a high disease burden at the time of relapse were associated with poorer treatment outcomes. In contrast, patients with a low disease burden and a longer interval between alloHSCT and relapse showed significantly better response and survival. The results were particularly favorable with preemptive therapy for molecular relapse or MRD positivity (51). **Table 1**.

### 3.5 Decitabine and DLI

There are only isolated reports on the use of decitabine as monotherapy without DLI for the treatment of relapse following alloHSCT; however, these are not significant due to the small number of patients (52). As part of a retrospective multicenter study, we enrolled 36 patients from 6 German centers with predominantly hematologic relapse (29 AML, 7 MDS). Patients received a median of 2 cycles of decitabine; 61% also received DLI. The ORR was 25%, including 6 CRs (17%) and 3 PRs (8%). The median duration of CR was 10 months, the 2-year OS rate was 11%; the incidence of acute and chronic GvHD was 19% and 5%, respectively. More than half had already received other salvage regimens; 16 patients had even previously received azacytidine (53). Another, smaller single-center series investigated decitabine plus DLI in 26 patients with predominantly AML/MDS and hematologic relapse following alloHSCT. Here, the strategy was more of a “bridge-to-second-alloHSCT” approach. With complete remissions (CR/Cri) in 15% (4/26) and partial remissions, as well as stable disease in 4% (1/26) and 58% (15/26), respectively, this report supports the feasibility of decitabine/DLI even in cases of more proliferative relapse (54). The evidence base for decitabine is clearly weaker than for azacytidine. Azacytidine ± DLI is the standard of care, while decitabine ± DLI remains more of a backup or salvage-after-azacytidine strategy. **Table 2**.

**Table 2:** Key studies on Decitabine +/- DLI for the treatment of relapse after alloHSCT.

Author	Year	Disease	Therapy**	Number	Response	Main findings/conclusions
Ganguly (52)	2013	AML/MDS	Decitabine + DLI (40%)	8	ORR 63% CR 63%	Well tolerated, better response in early relapse.
Schroede (53)r	2018	AML/MDS	Decitabine + DLI (61%)	36	ORR 25% CR 17%	Responses also seen in salvage after failure of Azacytidine, low rates of GvHD.
Sommer (54)	2018	AML/MDS	Decitabine + DLI (69%)	26	ORR 19% CR 15%	Additional 58% stable disease, feasibla as bridge to 2^nd^ alloHSCT.

**Table 2** shows details of the most important studies in Decitabine +/DLI for the treatment of MDS and AML relapse after alloHSCT. * Major, major response; Stable, stable disease; ORR, overall response (CR/CRi + PR); CR, complete response (CR, CRi). ** (…%) % of patients who received DLI.

### 3.6 Azacytidine combination therapy

#### 3.6.1 Sorafenib

Sorafenib was the first tyrosine kinase inhibitor to be widely used in clinical practice for the treatment of FLT3-ITD-positive AML. Early on, the drug demonstrated significant activity in patients with relapsed or refractory disease, both before and after alloHSCT. A distinction must be made here between its use as relapse therapy versus as maintenance therapy (55–57). In addition to its direct antiproliferative effect on FLT3-ITD-mutated leukemia cells, preclinical studies and clinical observations suggest that immunological mechanisms could contribute significantly to the therapeutic effect, particularly following allogeneic transplantation. Therefore, the substance is of great interest as a maintenance therapy (58).

When used as monotherapy for the treatment of relapse following alloHSCT, response rates of approximately 30–45% have been reported in several studies. In individual cases, long-lasting molecular remissions lasting >5 years have been achieved (59, 60). Furthermore, there is a plausible biological rationale for a synergistic effect of sorafenib and azacytidine. A Phase II study demonstrated that the combination of both agents is feasible and clinically effective even in advanced, previously treated patients. The response rate was 46%, including 10 CRi (27%), 6 CRs (16%), and 1 PR (3%) (61).

Against this background, the combination of azacytidine, sorafenib, and DLI appeared to be an attractive approach for treating patients with relapsed FLT3-mutated AML following alloHSCT. In our own series, a total of eight patients were treated. This resulted in a complete remission rate of 50% (62). Other, smaller case series also reported similarly positive results (63–65). Furthermore, a Chinese study using a four-drug combination of sorafenib, venetoclax, azacytidine, and homoharringtonine achieved a remarkable complete remission rate of 76.5% in patients with relapsed disease. This study included patients with primary refractory (29.4%) and relapsed disease (70.6%), of whom 27.5% had relapsed after alloHSCT. Among these patients, the CR/CRi rate was 78.6% after just one cycle (66). Overall, the combination of azacytidine and sorafenib therefore represents an interesting therapeutic option for patients with FLT3-ITD-mutated AML in relapse following alloHSCT. Although combination therapies with newer FLT3 inhibitors are currently being tested outside the transplant setting, experience in the treatment of relapses following alloHSCT remains very limited (67). **Table 3**.

**Table 3:** Key studies on HMA combination therapy +/- DLI for the treatment of relapse after alloHSCT.

Author	Year	Disease	Therapy**	Number^#^	Response*	Main findings/conclusions
Rautenberg (62)	2017	FLT3-ITD AML	Azacytidine + Sorafenib +DLI (75%)	8	ORR 50% CR 50%	Combination is feasible, high response rate, long lasting responses possible.
Sid (65)	2017	FLT3-ITD AML	Azacytidine + Sorafenib +DLI (0%)	5	ORR 60% CR 60%	Combination feasible, high response rate.
Yu (66)	2014	FLT3-ITD AML	Azacytidine + Sorafenib + Venetoclax + HO + DLI (79%)	51 (14)	ORR 82% CR 77%	Well tolerated, response rates similar in primary refractory, or relapsed disease.
Craddock (68)	2019	AML	Azacytidine + Lenalidomide +DLI (0%)	29	ORR 47% CR 40%	MTD was 25mg. no increased GvHD.
Schroeder (69)	2023	AML/MDS/CMML	Azacytidine + Lenalidomide +DLI (68%)	50	ORR 56% CR 59%	High response rate in molecular and hematologic relapse, no increased GvHD.
Schuler (73)	2021	AML/MDS	Azacytidine or Decitabine + Venetoclax +DLI (37%)	32	ORR 47% CR 37%	ORR higher in 1^st^ relapse (86%) than in advanced disease, high hematotoxicity.
Cheng (74)	2025	AML	Azacytidine + Venetoclax	53	ORR NA CR 57%	Higher efficacy and better tolerated that intensive chemotherapy.
Ionescu (76)	2024	AML	Azacytidine or Decitabine + Venetoclax +DLI (11%)	72	ORR NA CR 48%	Better response in Patients with NPM1 and IDH1/2 mutations, high hematotoxicity.
Joshi (77)	2021	AML	Azacytidine or Decitabine + Venetoclax +DLI	29	ORR 38% CR 28%	Active also with adverse mutations (TP53) and extramedullary relapse.
Venugopal (89)	2022	IDH2 AML	Azacytidine + Enasidenib (+Venetoclax) +DLI (%)	19 (5)	ORR 61% CR 61%	Combination was well tolerated, the addition of Venetoclax seems to improve results.

**Table 3** shows details of the most important studies in HMA combination regimens +/- DLI for the treatment of MDS and AML relapse after alloHSCT. * Major, major response; Stable, stable disease; ORR, overall response (CR/CRi + PR); CR, complete response (CR, CRi). ** (…%) % of patients who received DLI.# (…) number of patients who had relapse after alloHSCT. HO, homoharringtonine.

#### 3.6.2 Lenalidomide

Alongside sorafenib, lenalidomide is historically one of the first combination partners for azacytidine to be clinically targeted for the treatment of MDS and AML relapses following alloHSCT. As early as 2019, Craddock et al. published a Phase I/II study on this combination. The study included a total of 29 patients and followed a classic dose-escalation design with a subsequent Phase II expansion. The aim of the study was to determine the maximum tolerated dose of lenalidomide in combination with azacytidine at the standard dose of 75 mg/m² administered on seven consecutive days. The lenalidomide dose was gradually increased in cohorts, starting at 5 mg and progressing to 10 mg, 15 mg, and finally 25 mg. A dose of 25 mg of lenalidomide was ultimately identified as the maximum tolerated dose. Dose-limiting toxicities at this dose level included fatigue, rash, and elevated transaminases. Overall, 7 of 15 evaluable patients (47%) demonstrated a response defined as a major clinical response, and 6 achieved complete remission (CR/CRi). The predominant adverse effects were cytopenias and infections. There was no increased incidence of GvHD. Concurrently, immunological correlative studies on T-cell function were conducted. However, these studies revealed no relevant change in the production of interferon-γ or TNF-α, nor any significant influence of the therapy on markers of T-cell exhaustion (68).

In the prospective AZALENA study, in contrast to early dose-escalation trials, we pursued the concept of long-term, low-dose lenalidomide therapy in order to therapeutically exploit the substance’s immunomodulatory properties in particular, in addition to azacytidine. A total of 50 patients with AML (n=23), MDS (n=24), or CMML (n=3) with relapse following alloHSCT were treated. Hematologic relapse of the underlying disease was present in 48%, whereas 52% of patients had molecular relapse. This reflects the increasing trend in recent years toward early MRD-based diagnosis and treatment of relapses following alloHSCT.

Fourteen patients received 2.5 mg and 36 patients received 5 mg of lenalidomide on days 1–21 in addition to azacytidine (75 mg/m² on days 1–7). DLI was scheduled in ascending doses after cycles 4, 6, and 8 (**Figure 1**). Patients received a median of 7 (1–8) treatment cycles; 68% received at least one DLI. The overall response rate was 56%, with 25 patients (50%) achieving complete remission, which was sustained in 80% of cases. It is noteworthy that approximately half of the complete remissions were achieved prior to the first DLI, while the remaining remissions occurred after administration of donor lymphocytes. Interestingly, CR rates did not differ between patients treated for molecular relapse and those in whom therapy was initiated for hematologic relapse (56% vs. 44%; P = 0.778), nor between patients with early and late relapse, or between the two dosage levels (2.5 and 5 mg lenalidomide). Overall, we were able to demonstrate that lenalidomide at a dose of 5 mg can be safely administered in addition to azacytidine and DLI without significantly increasing hematotoxicity or the risk of GvHD (69). **Table 3**.

#### 3.6.3 Venetoclax

The combination of HMA and venetoclax (VEN) is the most important combination strategy, which has been extensively studied and used in recent years. However, it is not yet clear whether it can replace the current standard of care, azacytidine plus DLI. Initially, the combination showed promising results with high response rates in relapsed cases following conventional chemotherapy (70–72).

An early German multicenter analysis included 32 AML/MDS patients from 11 centers with relapse following alloHSCT. Venetoclax was combined with azacytidine (n=13) or decitabine (n=19); 11 patients additionally received DLI. The ORR was 47%. Therapy was particularly effective in cases of molecular relapse (ORR 67%) and as first-line relapse therapy (ORR 86%). Although the study was small and the regimens heterogeneous, it consistently demonstrated higher activity than classic HMA monotherapy and, at the same time, higher hematotoxicity (73).

More recent data confirm this impression, even when compared to conventional relapse therapy. In a retrospective cohort of 106 patients (53 HMA/VEN versus 53 chemotherapy) treated for relapse after alloHSCT, HMA/VEN was associated with longer median overall survival (12.6 versus 5.8 months) and longer relapse-free survival (9.1 versus 4.3 months) compared to conventional, intensive chemotherapy. At the same time, Grade 3/4 neutropenia (35.8% vs. 64.2%), Grade 3/4 thrombocytopenia (39.6% vs. 71.7%), and sepsis (11.3% vs. 32.1%) were less common with HMA/VEN. Although still retrospective, this is a clinically compelling result that goes beyond the classic aza/DLI approach (74). In first-line therapy, it has been shown that patients with myeloid neoplasms harboring mutations in IDH1, IDH2, NPM1, or DDX41, in particular, respond exceptionally well to the combination of hypomethylating agents (HMA) and venetoclax. These molecular subgroups are associated with high remission rates and more frequent MRD negativity. However, so far only scarce retrospective data has reported increased response rates in patients with NPM1 or IDH 1 or 2 mutations treated for relapse following alloHSCT. Therefore, the predictive value of these mutations for response to HMA/VEN in this specific clinical context cannot yet be universally assessed (75–78).

A meta-analysis of 13 studies involving 337 AML patients following HSCT showed a consistent trend of high efficacy but relevant toxicity after alloHSCT. In this systematic review presented at ASH2024, venetoclax was administered to all patients in combination with HMA, with azacytidine in 62% of patients and with decitabine in 38% of patients. The median time to relapse after alloHSCT was approximately 7 months. DLI was used in 27% of patients. The pooled overall response rate was 52% (95% CI 40–64%), and the pooled complete remission rate (CR/CRi) was 39% (95% CI 28–50%). With a median follow-up of 15.3 months, median survival was 8.6 months. However, this meta-analysis also highlights the high toxicity of HMA/VEN, including neutropenia (73% grade III–IV, 95% CI 63–82), thrombocytopenia (71% Grade III–IV, 95% CI 62–79%), and infections (31% Grade III–IV, 95% CI 14–56%). Accordingly, the pooled mortality rate was high at 29% (95% CI, 9–64) (79). Therefore, it remains unclear whether the high response rates can ultimately deliver better long-term remissions compared to the standard treatment of azacytidine and DLI. Given the pronounced and often dose-limiting hematotoxicity of conventional combination regimens using azacytidine or decitabine with venetoclax, alternative strategies involving reduced dosing as well as metronomic low-dose regimens have been developed in recent years. Initial clinical studies in treatment-naive patients with AML suggest that these approaches can achieve response rates close to those of established standard regimens despite significantly lower exposure to the drugs used. Given the often limited hematopoietic reserve and increased susceptibility to infection in patients with relapse following alloHSCT, this approach appears particularly attractive in this setting. It is therefore conceivable that reduced-dose or metronomic HMA/VEN regimens may also provide a more favorable balance between efficacy and toxicity in this setting (80–83). **Table 3**.

#### 3.6.4 IDH inhibitors

More recently, additional targeted therapies have been studied in combination with hypomethylating agents, particularly azacytidine, for the treatment of AML and MDS. In particular, the IDH1 and IDH2 inhibitors ivosidenib and enasidenib are considered promising combination partners. In a Phase I/II study, DiNardo et al. demonstrated that the combination of ivosidenib and azacytidine was well tolerated in patients with newly diagnosed IDH1-mutated AML who were not suitable for intensive induction therapy. The combination was well tolerated and achieved high response rates (ORR 78.3%, CR/CRi 60.9%) (84, 85). The combination of azacytidine and ivosidenib was later on investigated in the randomized Phase 3 AGILE trial. Here, the combination demonstrated improved remission rates (CR/CRi 54% vs. 16%) compared to azacytidine alone, as well as prolonged event-free and overall survival (86, 87).

However, only limited data are currently available regarding its use following alloHSCT. In the French multicenter IDALLO study of relapsed or refractory IDH1-mutated AML, monotherapy with ivosidenib demonstrated a response rate of 41% (CR 36%) (88). Venugopal et al. reported, among other findings, on a small subgroup of patients with relapse after alloHSCT in whom clinical activity was also observed with the combination therapy of azacytidine, enasidenib, and venetoclax, although efficacy decreased with an increasing number of prior lines of therapy (89). Overall, the combination of IDH inhibitors and azacytidine appears particularly attractive for use after allogeneic transplantation in myeloid diseases with IDH mutations due to potentially synergistic mechanisms of action.

Similar considerations apply to MENIN inhibitors, which have already demonstrated clinical efficacy as monotherapy in AML with KMT2A rearrangements or NPM1 mutations (90, 91). Combinations with azacytidine also appear biologically plausible in this context. Initial Phase I/II studies with triple combinations of azacytidine, venetoclax, and revumenib have already demonstrated very high response rates (ORR 88%, CD/CRi 81%) and good tolerability in the first-line setting (92). However, clinical data for use in relapsed disease following alloHSCT are currently lacking. **Table 3**.

## 4 Discussion and conclusion

Over the past two decades, hypomethylating agents—particularly azacytidine—have evolved from an experimental therapeutic approach to the current therapeutic backbone for the treatment of relapsed MDS and AML following alloHSCT. Nevertheless, this treatment paradigm remains dynamic and may further evolve with the increasing availability of targeted agents, novel immunotherapeutic approaches, and molecularly guided combination strategies. In particular, the RELAZA studies from Dresden demonstrated that monotherapy with azacytidine in molecular relapse is capable of delaying the onset of hematologic relapse over a longer period of time. Nevertheless, without further cellular immunotherapy, nearly all patients eventually experience hematologic relapse (35–37). This underscores the critical importance of immunological mechanisms—particularly through the administration of donor lymphocytes (DLI) or a second alloHSCT—for cure.

As a consequence, the combination of azacytidine and DLI currently represents the most widely used salvage strategy in clinical practice. Whether this approach will remain the preferred therapeutic backbone in the era of molecularly targeted therapies and novel immunotherapeutic combinations remains an open question. However, based on clinical studies to date, the respective contributions of hypomethylating therapy and cellular immunotherapy to achieving remission can only be distinguished from one another to a limited extent. In the AZARELA study, the first DLI was administered after only two cycles of azacytidine. Therefore, cytotoxic and immunological therapeutic effects were closely linked (42). In the subsequently conducted AZALENA study, a deliberate effort was made to administer the first DLI only after the fourth treatment cycle (**Figure 1**). Notably, approximately half of the remissions occurred before the first administration of donor lymphocytes. This finding suggests that both, pharmacological therapy with hypomethylating agents and cellular immunotherapy make a significant and complementary contribution to achieving and maintaining remission (69).

**Figure 3:**
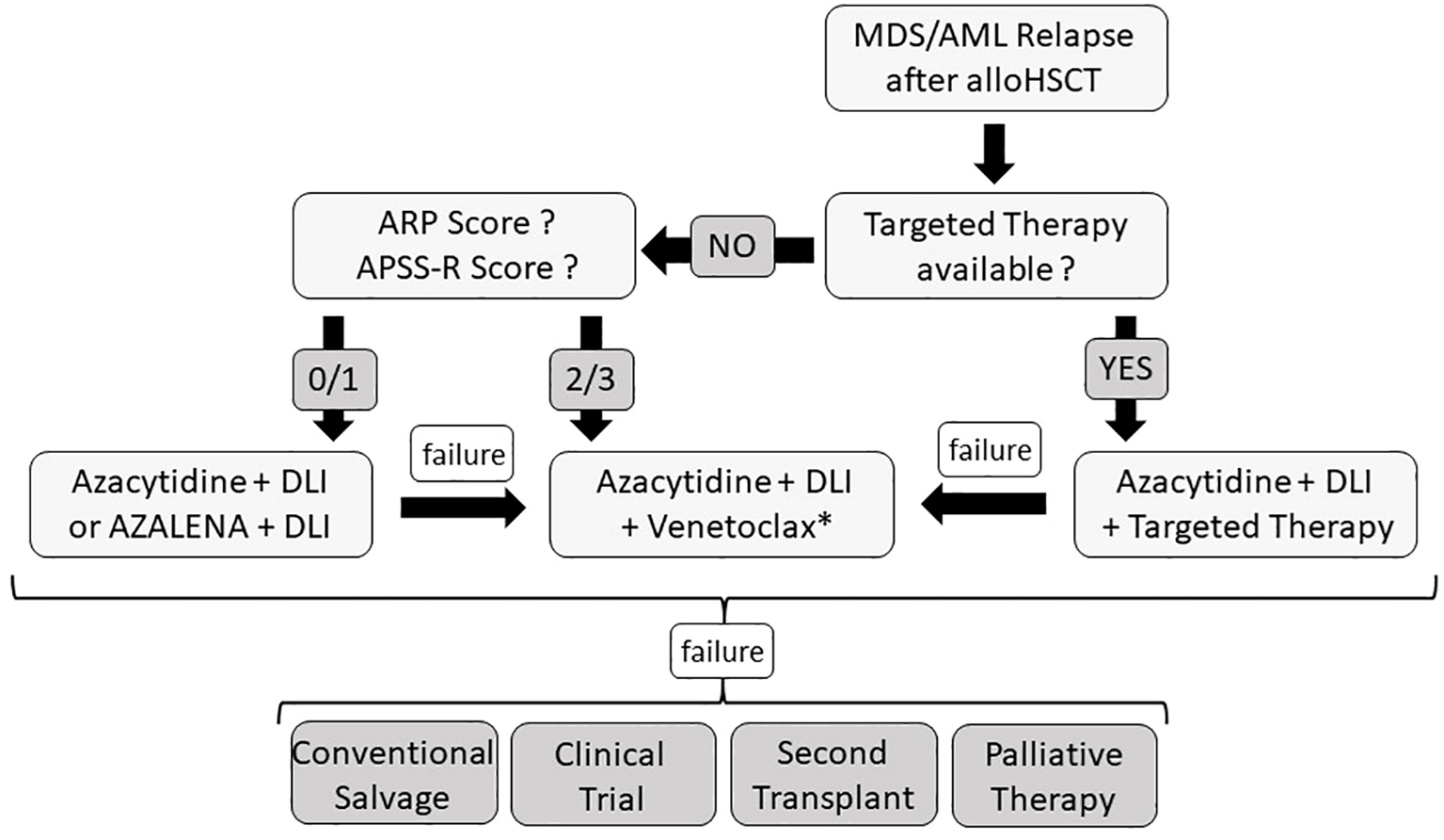
Suggested recommendations for selecting HMA based therapy for relapsed MDS and AML following alloHSCT. If targeted therapy is available, a combination of Azacytidine and the targeted therapy (+DLI) may be be preferred. *Also patients with distinct molecular features (IDH1/2, NPM1, DDX41) seem to benefit most from HMA + Venetoclax (+DLI). If this is not available, patients with a low ARP or APSS-R score can be treated with Azacytidine alone (+DLI) or in combination with Lenalidomide (+DLI). Response rates are high in these cases (44, 47). Patients with higher scores have a poorer prognosis and may be treated with the more intensive combination therapy including Azacytidine or Decitabine and Venetoclax (+DLI). It is not known which patients in CR require a second consolidating alloHSCT.

In recent years, various combination therapies based on azacytidine have been clinically investigated (51). Here, targeted therapeutic approaches—for example, in FLT3-, IDH1-, or IDH2-mutated AML and MDS —can be distinguished from combinations with a more broad-spectrum effect, such as azacytidine plus lenalidomide or venetoclax. In particular, combinations with molecularly targeted agents and with venetoclax have shown high response rates in the corresponding molecularly defined entities. Consequently, these are most likely the preferred treatment options today, provided that the relevant mutation is detectable at relapse and the targeted therapy is available [**Figure 3** (62, 84)]. In this context, repeat molecular genetic testing at relapse is of central importance, as the mutation profile can often change between initial diagnosis and relapse (11, 93).

Beyond their therapeutic implications, molecular abnormalities may also influence the likelihood of achieving durable responses following HMA-based salvage approaches after alloHSCT. In particular, TP53 mutations and other adverse molecular lesions associated with genomic instability and therapy resistance are consistently linked to an increased risk of relapse and inferior survival following alloHSCT (75–78, 94). Furthermore, recent studies have demonstrated that the molecular landscape at relapse frequently evolves through clonal selection and the acquisition of additional genetic alterations, underscoring the importance of repeat molecular profiling to guide therapeutic decision-making (11). Although robust data specifically evaluating the predictive impact of individual mutations on response to HMA ± DLI remain limited, molecular disease biology is increasingly recognized as a major determinant of relapse kinetics and long-term treatment outcomes after transplantation. Collectively, these observations suggest that future prognostic models, including refinements of the APR and APSS-R scores, may benefit from incorporating molecular genetic risk factors to enable more individualized therapeutic decision-making.

The more broadly targeted combinations also demonstrate marked clinical activity. Azacytidine + lenalidomide proved effective particularly in early molecular or cytogenetic disease recurrence, but also demonstrated good activity in hematological relapse. However, the corresponding studies predominantly included patients without highly proliferative acute leukemias, specifically excluding patients with FLT3-mutated AML (68, 69). In contrast, venetoclax-based combinations demonstrated the best response rates in patients with NPM1 or IDH mutated disease, but also in more advanced relapse situations (95). However, HMA/VEN combinations are frequently associated with pronounced myelosuppression and an increased risk of infection. Therefore low dose, metronomic schemes have been developed that still have to be tested in the post allo setting. Overall, the available data suggest that both, the disease biology and the dynamics of relapse, as well as the tailored selection of combination partners, are crucial for the success of the respective salvage strategy (**Figure 3**).

Despite the strong biological rationale for combining hypomethylating agents with immunotherapeutic or immune-modulating approaches, many of these strategies have failed to demonstrate a clear clinical benefit even in earlier treatment lines and therefore have not been established in the post-transplant relapse setting. This includes immune checkpoint inhibition, macrophage checkpoint inhibition, and TIM-3 targeting. For example, the addition of durvalumab to azacitidine failed to improve survival in patients with higher-risk MDS despite encouraging preclinical data (27). Similarly, the anti-CD47 antibody magrolimab initially showed promising activity in combination with azacitidine in early phase studies in higher-risk MDS and AML (96), but subsequent randomized phase III studies failed to confirm this benefit. In particular, the ENHANCE-2 study in TP53-mutated AML and the ENHANCE-3 study in patients with AML unfit for intensive chemotherapy did not demonstrate an improvement in survival outcomes following the addition of magrolimab to azacitidine-based regimens (97). Likewise, the randomized STIMULUS-MDS1 trial failed to demonstrate significant improvements in complete remission rates or progression-free survival with the addition of the anti-TIM-3 antibody sabatolimab to hypomethylating agents in higher-risk MDS (98). Taken together, these findings illustrate that HMA-induced immunomodulation does not necessarily translate into improved anti-leukemic efficacy. The highly heterogeneous immune landscape after alloHSCT, together with differences in disease biology, disease burden, prior therapies, and the timing of intervention, likely contributes to these inconsistent outcomes. Therefore, future studies should focus on biologically guided patient selection and the identification of predictive biomarkers to optimize HMA-based immunotherapeutic strategies.

Although AML and MDS share several biological features and are often treated with similar post-transplant strategies, important disease-specific differences should be considered. AML relapses are frequently characterized by rapid disease kinetics, a higher proliferative capacity, and a shorter interval from alloHSCT to relapse, often necessitating immediate therapeutic intervention and limiting the time window for immunological approaches to unfold (43, 44). In contrast, MDS relapses more commonly present as molecular or cytogenetic recurrence with slower progression kinetics, thereby offering a broader opportunity for MRD-guided interventions and sequential immunotherapeutic strategies (44, 47). Consistent with this concept, several studies have demonstrated higher response rates and superior survival outcomes in patients with MDS compared with AML following treatment with azacytidine ± DLI (43, 44, 47, 48). Furthermore, the optimal timing and integration of donor lymphocyte infusions may differ between both entities, as patients with slowly evolving MDS relapse may benefit from delayed and repeated immunological interventions, whereas rapidly progressive AML often requires more immediate cytoreductive approaches (43, 44). Future studies should therefore increasingly address AML and MDS separately and aim to develop disease-specific therapeutic algorithms for post-transplant relapse management.

To improve risk stratification of patients with relapsed AML or MDS following alloHSCT, several prognostic scores have been developed in recent years. These scores take into account, in particular, disease dynamics—measured by the time of recurrence after transplantation—as well as disease burden at the time of relapse diagnosis. The British APR score analyzed exclusively hematologic relapses and distinguished them based on a bone marrow blast count of 20% (44). In contrast, the APSS-R score differentiates between molecular and hematologic relapse and additionally incorporates the timing of relapse into the prognostic assessment. Another distinctive feature of the APSS-R score is that, for MDS, it also takes into account the type and duration of prior therapy before allogeneic transplantation [**Figure 2** (47, 48)].

Particularly in patients with higher-risk MDS and adverse molecular features, prolonged pre-transplant treatment may contribute to the selection of therapy-resistant clones and increase the likelihood of relapse after alloHSCT (12, 99). However, the rationale for upfront transplantation without prior therapy should be interpreted in a nuanced manner. Emerging data suggest that minimizing time-to-transplant is of major importance in higher-risk MDS, as prolonged bridging strategies may delay transplantation, increase the risk of disease progression, infectious complications, and loss of transplant eligibility (100). Studies such as VIDAZA-ALLO and recent analyses evaluating optimal time-to-transplant indicate that extended pre-transplant treatment does not necessarily improve post-transplant outcomes and may even be detrimental in selected patients (100, 101). Therefore, therapeutic decisions should aim to balance disease control with timely referral to transplantation, avoiding unnecessary delays while individualizing treatment according to disease biology, comorbidities, donor availability, and the patient’s overall fitness for alloHSCT. In this context, the prevention and management of post-transplant relapse begins already at the time of initial diagnosis and requires rational sequencing of therapies to minimize the development of treatment-resistant disease (102–105).

The question remains open as to whether therapy with hypomethylating agents in combination with donor lymphocyte infusions and, if necessary, other agents is sufficient for long-term disease control or cure and which patients require a consolidating second alloHSCT. Clinical observations show that a significant proportion of patients can achieve long-lasting remissions without a second transplant. Importantly, however, a second alloHSCT remains a potentially curative option for selected patients who relapse after the first transplantation, even in the absence of hematologic complete remission following HMA ± DLI-based salvage therapies (106, 107). Several studies have demonstrated that patients relapsing more than six months after the initial alloHSCT have a substantially more favorable prognosis and may achieve meaningful long-term survival after a second transplantation, with reported overall survival rates of approximately 40% at one year and around 25% at five years (106, 107). Notably, outcomes after a second alloHSCT appear to depend not only on remission status but also on patient fitness, comorbidity burden, and transplant eligibility (106, 107). Therefore, the possibility of a second alloHSCT should remain an integral component of therapeutic decision-making in patients with post-transplant relapse, including those with persistent active disease. Consequently, the identification of prognostic factors that can be used to individually assess the necessity of a second alloHSCT represents a key future research goal.

Performing a second allogeneic stem cell transplant is associated with significant transplant-related morbidity and mortality and should therefore, if possible, be reserved for those patients whose likelihood of long-term disease control through HMA-based salvage therapies with DLI is low (108). However, we and others were able to demonstrate that even after failure of HMA-based relapse therapy, a salvage transplant can still be performed successfully (2, 106, 107). This suggests that a risk-adapted strategy could be appropriate, in which a second alloHSCT is selectively used for those patients in whom the prospect of durable remission through HMA-based therapeutic approaches alone is insufficient (**Figure 3**).
